# A Review of Family-Based Tests for Linkage Disequilibrium between a Quantitative Trait and a Genetic Marker

**DOI:** 10.1371/journal.pgen.1000180

**Published:** 2008-09-26

**Authors:** Warren J. Ewens, Mingyao Li, Richard S. Spielman

**Affiliations:** 1 Department of Biology, University of Pennsylvania, Philadelphia, Pennsylvania, United States of America; 2 Department of Biostatistics and Epidemiology, University of Pennsylvania, Philadelphia, Pennsylvania, United States of America; 3 Department of Genetics, University of Pennsylvania, Philadelphia, Pennsylvania, United States of America; University of Alabama at Birmingham, United States of America

## Abstract

Quantitative trait transmission/disequilibrium tests (quantitative TDTs) are commonly used in family-based genetic association studies of quantitative traits. Despite the availability of various quantitative TDTs, some users are not aware of the properties of these tests and the relationships between them. This review aims at outlining the broad features of the various quantitative TDT procedures carried out in the frequently used QTDT and FBAT packages. Specifically, we discuss the “Rabinowitz” and the “Monks-Kaplan” procedures, as well as the various “Abecasis” and “Allison” regression-based procedures. We focus on the models assumed in these tests and the relationships between them. Moreover, we discuss what hypotheses are tested by the various quantitative TDTs, what testing procedures are best suited to various forms of data, and whether the regression-based tests overcome population stratification problems. Finally, we comment on power considerations in the choice of the test to be used. We hope this brief review will shed light on the similarities and differences of the various quantitative TDTs.

## Introduction

This review aims at outlining the broad features of various frequently used quantitative transmission/disequilibrium tests (quantitative TDTs). We focus on describing the models assumed in these tests and the relationships between the tests. It is impossible in a brief review to describe and compare the great variety of quantitative TDT procedures to be found in the literature and that are available in computer packages, because some of these procedures involve quite complex forms of data and a sophisticated statistical analysis. We have therefore deliberately restricted the scope of this review by considering only one simple form of data—namely, family trios—that is families with a mother, a father, and exactly one child. Further, we consider only the basic features of the procedures that we discuss. Even with these limitations, a number of interesting questions arise, some of which we raise but do not answer. We leave a deeper analysis of the procedures discussed here, and an analysis of the more complex procedures that we do not consider, to another occasion.

The aim of the original qualitative TDT procedure [1] was to test for linkage (and linkage disequilibrium) between a marker locus and a disease locus in a way that overcomes problems arising from potential population stratification. We assume the same null hypothesis for the quantitative TDT procedures considered here (see below for details). For convenience, we focus here on quantitative TDT procedures carried out in the frequently used FBAT (http://biosun1.harvard.edu/~fbat/fbat.htm) and QTDT (http://www.sph.umich.edu/csg/abecasis/QTDT/) packages. Specifically we discuss the “Rabinowitz” [2] and the “Monks-Kaplan” [3] procedures, as well as various “Abecasis” [4] and “Allison” [5] regression-based procedures, when applied specifically to family trio data. The questions addressed in this review are as follows:

- What are the properties of the various Allison/Abecasis regression-based tests and the Rabinowitz and the Monks-Kaplan tests?
- What is the relation between the regression-based tests and the Rabinowitz and Monks-Kaplan tests?
- What hypotheses are tested by the various quantitative TDT procedures that we describe?
- What testing procedures are best suited to various forms of data?
- Do the regression-based tests that we describe overcome population stratification problems?
- What power considerations arise in the choice of the test to be used?

We note that some of these issues were previously considered by Lange et al. [6] from a different viewpoint.

## Notation and Data

The notation used in the various quantitative TDT papers on which our comments are based is not consistent from one author to another, and we adopt a unifying notation that is loosely based on that of these papers. In accordance with standard statistical practice, we use upper case notation for random variables and the corresponding lower case notation for the observed values of these random variables. To focus on the main points in this expository review, we assume a specific (and restricted) form of data. We assume that the data concern a marker locus “*A*,” having two possible alleles, denoted by *A* and *a*, and consist of information on *n* family trios, with complete marker locus genotype information on the two parents and the child in each trio. The value of the quantitative trait of interest is known for the child in each trio but not for the parents. We assume, in line with the original qualitative TDT, that all parental mating types are informative (i.e., contain at least one *Aa* parent). The observed number of *A* alleles in the child in trio *i* is denoted by *x_i_* (*i* = 1, 2,…, *n*), and the observed value of the quantitative trait of interest in the child in trio *i* is denoted by *y_i_*.

We do not consider here the extent to which the comments made below carry over to data other than those described above, for example cases where several children are observed in each family, where parental phenotype information is available, and where the data contain families with noninformative mating types. We restrict our analysis in this way so as to highlight the main features of the testing procedures that we discuss without getting into the analyses required for forms of data more complicated than those we consider.

The null hypothesis tested is “no linkage (or no linkage disequilibrium) between the marker locus and a locus involved with the quantitative trait.” Under this null hypothesis, the mean number of *A* alleles, *X_i_*, in the child in trio *i* will depend on the parental mating type, being 0.5 if it is *aa*×*Aa*, 1.0 if it is *Aa*×*Aa*, and 1.5 if it is *AA*×*Aa*. The null hypothesis variance 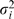 of *X_i_* also depends on the parental mating type, being 0.25 if it is *aa*×*Aa* or *AA*×*Aa* and 0.5 if it is *Aa*×*Aa*. We frequently use the convenient Abecasis [5] notation *W_i_* (“within family”) to describe *X_i_* minus its null hypothesis mean as computed from the mating type in trio *i*. The null hypothesis mean of *W_i_* is zero and the null hypothesis variance of *W_i_* is the same as that for *X_i_*, and depends on the mating type in trio *i*. When discussing the typical family we drop the suffix *i* and use the generic notation *W*, *w*, *X*, *x*, *Y*, and *y*.

## Quantitative TDT Models

In this section, we describe in algebraic terms the various quantitative TDT procedures outlined above and address questions raised in the Introduction.

### 

#### Properties of various quantitative TDT procedures

We start with five Allison and Abecasis “regression-based” procedures. (The Abecasis “total” test is not a TDT test [as is indicated in the QTDT package documentation], so we ignore it.) These all assume a regression model where the mean of the phenotype *Y_i_* of the child in trio *i* depends on the actual value *w_i_* for that child, often along with other information, for example, the parental mating type of trio *i*. More precisely, the five models that we consider are as follows:

1. The Abecasis “within only” model, denoted here Ab-Wthn. In this model, *Y* is assumed to depend only on the value of *w*, the observed difference between the number of *A* alleles in any child and the null hypothesis mean of this number, given the parental mating type for this child.
2. The Abecasis “orthogonal” model, denoted here Ab-Orth. In this model, *Y* is assumed to depend on *w* and also a linear term describing parental mating type.
3. The Abecasis “dominance” model, denoted here by Ab-Dom. This model generalizes Ab-Orth in that *Y* is assumed also to depend on whether the child in any trio is a homozygote or a heterozygote.
4. The first Allison model, denoted here Al-Lin. This is a “general/linear” model, where *Y* is assumed to depend on parental mating type in an unspecified way and also on *w*.
5. The second Allison model (his TDTQ5), denoted here Al-Quad. This is a “general/quadratic” model, and extends Al-Lin by assuming that *Y* depends also on *w* ^2^. In algebraic terms, the assumptions for the “full,” or alternative, hypothesis case of these models can be written in terms of the three mating types considered as follows:

*Abecasis* “*within only*” *model* (*Ab-Wthn*).

For all parental mating types: *Y* = *μ*+*β*_1_ *w*+*E*.

*Abecasis* “*orthogonal*” *model* (*Ab-Orth*).

For *aa*×*Aa* parental mating type: *Y* = *μ*+*β*_1_ *w*+*E*;
For *Aa*×*Aa* parental mating type: *Y* = *μ*+*α*+*β*_1_ *w*+*E*;
For *AA*×*Aa* parental mating type: *Y* = *μ*+2*α*+*β*_1_ *w*+*E*.

*Abecasis* “*dominance*” *model* (*Ab-Dom*).

For *aa*×*Aa* parental mating type: *Y* = *μ*+*β*_1_ *w*+*γd*+*E*;
For *Aa*×*Aa* parental mating type: *Y* = *μ*+*α*+*β*_1_ *w*+*γd*+*E*;
For *AA*×*Aa* parental mating type: *Y* = *μ*+2*α*+*β*_1_ *w*+*γd*+*E*.

(In this model, *d* = −1 for a homozygous child and +1 for a heterozygous child, and corresponds to *W_d_* in the QTDT package documentation. For the data that we consider, the *B_d_* term in QTDT package documentation for this model is a constant across the three mating types, and is thus absorbed into the constant *μ*.)

*Allison* “*general*/*linear*” *model* (*Al-Lin*).

For *aa*×*Aa* parental mating type: *Y* = *μ*+*β*_1_*x+*E*;
For *Aa*×*Aa* parental mating type: *Y* = *μ*+*α*_1_+*β*_1_*x+*E*;
For *AA*×*Aa* parental mating type: *Y* = *μ*+*α*_2_+*β*_1_*x+*E*.

*Allison* “*general*/*quadratic*” *model* (*Al-Quad*).

For *aa*×*Aa* parental mating type: *Y* = *μ*+*β*_1_*x+*β*_2_*x ^2^+*E*;
For *Aa*×*Aa* parental mating type: *Y* = *μ*+*α*_1_+*β*_1_*x+*β*_2_*x ^2^+*E*;
For *AA*×*Aa* parental mating type: *Y* = *μ*+*α*_2_+β_1_*x+*β*_2_*x ^2^+*E*.

In all four models Greek symbols describe unknown parameters and *E* is a random residual term having mean zero and (unknown) variance 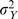. Because of the relations *w* = *x*−1/2 for *Aa*×*aa* matings, *w* = *x*−1 for *Aa*×*Aa* matings, and *w* = *x*−3/2 for *Aa*×*AA* matings, Al-Lin and Al-Quad can be re-written conveniently as:

*Allison* “*general*/*linear*” *model* (*Al-Lin*).

For *aa*×*Aa* parental mating type: *Y* = *μ*_1_+*β*_1_ *w*+*E*;
For *Aa*×*Aa* parental mating type: *Y* = *μ*_2_+*β*_1_ *w*+*E*;
For *AA*×*Aa* parental mating type: *Y* = *μ*_3_+*β*_1_ *w*+*E*.

*Allison* “*general*/*quadratic*” *model* (*Al-Quad*).

For *aa*×*Aa* parental mating type: *Y* = *μ*_1_+(*β*_1_+*β*_2_)*w*+*β*_2_ *w* ^2^+*E*;
For *Aa*×*Aa* parental mating type: *Y* = *μ*_2_+(*β*_1_+2*β*_2_)*w*+*β*_2_ *w* ^2^+*E*;
For *AA*×*Aa* parental mating type: *Y* = *μ*_3_+(*β*_1_+3*β*_2_)*w*+*β*_2_ *w* ^2^+*E*.

The null hypothesis tested in the Ab-Wthn, Ab-Orth, and Al-Lin models is *β*_1_ = 0, and in the Ab-Dom model is *β*_1_ = *γ* = 0. The null hypothesis tested in the original Al-Quad model is 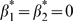, and this is equivalent to *β*_1_ = *β*_2_ = 0 in the re-written version above. The testing procedures in all five cases follow standard multiple regression methods, with mating type membership denoted with indicator variables. The null hypothesis model in each case removes a certain sum of squares for the phenotypic measurements in the children, and the full model removes a larger (or in rare cases, an equal) sum of squares. The difference between these two sums of squares forms the key component of the numerator of the *F* statistic used in all testing methods. This component is divided by the respective “model” degrees of freedom, which is equal to the number of extra parameters in each full model compared to the number in the corresponding null hypothesis model. This number takes the value 1 for Ab-Wthn, Ab-Orth, and Al-Lin and takes the value 2 for Ab-Dom and Al-Quad. This division by 2 tends to lead to smaller *F* ratios for Ab-Dom and Al-Quad, and thus to reduce power, and it is a trade-off against the increased generality of those models. This point is discussed further below.

The use of the *F* distribution to determine the significance of the observed value of the *F* statistic is appropriate only if the data have a normal distribution. For cases where the data are taken from one extreme tail of some distribution, for example very large values of the quantitative measurement, this might be an unreasonable assumption. This matter is discussed further below.

The three Abecasis models are nested, with Ab-Wthn being a special case of Ab-Orth, which in turn is a special case of Ab-Dom. Similarly Al-Lin is a special case of Al-Quad. Ab-Wthn and Ab-Orth are also special cases of Al-Lin. The nesting property is reflected in the residual degrees of freedom for the respective models: under the assumptions we have made concerning the data analyzed, the Ab-Wthn model has *n*−2 residual degrees of freedom, the Ab-Orth model has *n*−3 residual degrees of freedom, the Al-Lin and the Ab-Dom models have *n*−4 residual degrees of freedom, and the Al-Quad model has *n*−5 residual degrees of freedom.

There are regression-based models that are more general than those discussed above. A model more general than Ab-Dom and Al-Lin, and including these as particular cases, is:

“*General/dominance*” *model*.

For *aa*×*Aa* parental mating type: *Y* = *μ*_1_+*β*_1_ *w*+*γδ*+*E*;
For *Aa*×*Aa* parental mating type: *Y* = *μ*_2_+*β*_1_ *w*+*γδ*+*E*;
For *AA*×*Aa* parental mating type: *Y* = *μ*_3_+*β*_1_ *w*+*γδ*+*E*.

This model has *n*−5 residual degrees of freedom. A model more general than this, with *n*−6 residual degrees of freedom, and which includes all regression-based models described above, allows a term in *w* ^2^ as well as those in the “general/dominance” model. These more general models are not considered further here.

We can also consider testing procedures other than those described above. In particular, we suggest a modification of the Ab-Dom test, in which the null hypothesis is changed from the present *β*_1_ = *γ* = 0 to simply *β*_1_ = 0. This is for two reasons. First, it does not seem natural to test simultaneously the two hypotheses that no dominance phenotypic effects exist and that *y* does not depend on the transmission values *w*. Second, in testing the hypothesis *β*_1_ = 0 instead of *β*_1_ = *γ* = 0, one only has a single “model” degree of freedom, leading to increased power (compared to Ab-Dom) in testing for transmission effects.

We now describe the Rabinowitz [2] and Monks-Kaplan [3] procedures. In both of these procedures, the phenotype measurements *y*_1_, *y*_2_,…,*y_n_* in the children in the *n* trios are taken as given, and used as weights on the transmission random variables *W*_1_, *W*_2_,…,*W_n_*. This is in direct contrast to the Abecasis [4] and Allison [5] regression-based procedures, which take the *w_i_* as given and the phenotype measurements *Y*_1_, *Y*_2_, …, *Y_n_* as random variables. It is, however, more in line with the original qualitative TDT, which also uses *W*_1_, *W*_2_, …,*W_n_* as the random variables of interest.

For the data that we consider, Rabinowitz [2] defines 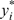 by 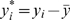, where *y̅* is the average of the *y_i_* values taken over the *n* children in the data. Given the observed values *w*_1_, *w*_2_,…, *w_n_* of *W*_1_, *W*_2_,…, *W_n_*, his test statistic *z* is
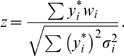
(1)In this expression, the sum (as with all sums in this article) is taken over *i* = 1, 2, …, *n*, and 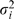 is as defined above. This statistic is based on the “within family” *w_i_* values because if this is done [2], the effects of population stratification are overcome. (This parallels a similar observation in the original qualitative TDT [1].) The Rabinowitz statistic is written as a *z* rather than a *t* because, with the 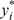 taken as given, the null hypothesis standard deviation of 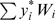
—the term in the denominator of Equation 1 —is known. Central limit theorem arguments then show that if *n* exceeds about 20, the Rabinowitz statistic has an approximate *N*(0, 1) distribution when the null hypothesis is true.

The Monks-Kaplan statistic is similar to the Rabinowitz statistic, being
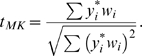
(2)The numerators in *z* and *t_MK_* are the same, but the denominator in the Monks-Kaplan statistic contains a standard deviation estimate rather than a known standard deviation. (This allows generalizations to handle data more complex than the data considered here.) It is written as a *t* statistic because of this fact.

#### Relationship between regression-based tests and Rabinowitz and Monks-Kaplan tests

The Rabinowitz and the Monks-Kaplan procedures differ from the Allison and the Abecasis procedures in various ways, of which we mention two. First, and most important, they regard the *W_i_* values as random variables with the *y_i_* values taken as given, whereas the Abecasis and Allison procedures regard the *Y_i_* values as random variables with the *w_i_* values taken as given. Second, unlike the Abecasis and Allison procedures, neither is explicitly based on regression models (see Laird et al. [7] for more details). Despite these differences, it is interesting to consider the hypothesis testing procedure in a “role-reversal” regression model of the form
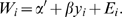
(3)This model, when compared to the Allison and Abecasis procedures, reverses the roles of *W* and *Y* in the regression. Because the Rabinowitz and Monks-Kaplan statistics are defined in terms of 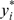 rather than *y_i_*, it is convenient to reformulate Equation 3 equivalently as
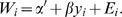
(4)The estimate of β in this regression is 
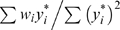
, and the standard regression *t* statistic testing for departures of *β* from zero is
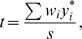
(5)where *s* is the usual regression estimate of the standard deviation of 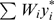. The Rabinowitz statistic (Equation 1) has the same numerator as that in Equation 5 but has, in the denominator, the known null hypothesis standard deviation of 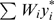 rather than a regression-based estimate of this standard deviation. The Monks-Kaplan statistic also has the same numerator as that in Equation 5, but has a standard deviation estimate in the denominator different from that in both Equation 1 and Equation 5.

Despite this similarity, there are essential differences between the Allison and Abecasis regression procedures and the Rabinowitz and Monks-Kaplan procedures. The Rabinowitz procedure, and in general all FBAT procedures, use “score statistics” based on the alleles transmitted to the children, conditional on the parental genotypes and the offspring phenotype. They are not explicitly based on regression models. Under the score test approach, the null hypothesis distribution of the test statistic is calculated directly from Mendel's laws. The test statistic thus has the correct distribution so long as these laws hold, regardless of any hypothetical model for the mean and variance of the offspring phenotype.

#### Hypotheses tested by various quantitative TDT procedures

There is an important difference between the hypothesis being tested by all the quantitative TDT procedures described above and the original qualitative TDT. The original TDT assesses whether the sum of the *w_i_* values differs significantly from zero. By contrast, none of the quantitative TDT procedures described above assess whether the *w_i_* values (or their weighted sum in the Rabinowitz and Monks-Kaplan procedures) differ significantly from zero. This can be seen from the fact that they are all unchanged if an arbitrary constant is added to the *w_i_* values. What they do test is whether there is significant change in the value of *w* as *y* changes (or of *y* as *w* changes). This is explicit in the regression procedures and also applies for the Rabinowitz and Monks-Kaplan procedures. We discuss this fact below in the context of the data that the investigator is analyzing. By contrast, it is the intercept in the estimated regression (Equation 4), namely *w̅*, that is directly comparable to the qualitative TDT statistic. We now show that, if the Rabinowitz approach of using the known variance of *W_i_* is taken, the test of whether this intercept is zero is identical to the original qualitative TDT procedure.

The standard regression test statistic of the null hypothesis *α* = 0 in the regression model (Equation 4) is the estimated intercept (in this case *w̅*) divided by the estimated standard deviation of *W̅*. In this case, the standard deviation of *W̅* is known, so the appropriate (*z*) test statistic is *w̅* divided by the standard deviation of *W̅*. It is equivalent and more convenient to use *z* ^2^ as test statistic, where *z* ^2^ can be written as 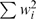 divided by the variance of 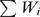. Under the null hypothesis, *z* ^2^ has an approximate chi-square distribution with one degree of freedom. We now calculate the value of *z* ^2^ in terms of transmission information.

We consider first the case of those trios having either *Aa*×*AA* or *Aa*×*aa* matings. If in any such trio the heterozygous parent transmits the *A* allele, the value of *w* for that trio is +1/2. We write the number of such trios as *m*_1_. If in any such trio the heterozygous parent transmits the *a* allele, the value of *w* for that trio is −1/2. We write the number of such trios as *m*_2_. The value of *w* in any trio where the parental mating type is *Aa*×*Aa* is +1 if both parents transmit the *A* allele, 0 if one parent transmits the *a* allele and the other parent transmits the *A* allele, and −1 if both transmit the *a* allele. We write the respective numbers of these trios as *m*_3_, *m*_4_, and *m*_5_. Thus 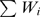 is *m*_1_/2−*m*_2_/2+*m*_3_−*m*_5_ = *m*_1_−*m*_2_+2*m*_3_−2*m*_5_/2. But this is just (*b*−*c*)/2, where *b* is the total number of transmissions of *A* from heterozygous parents and *c* is the total number of transmissions of *a* from heterozygous parents. The numerator in *z* ^2^ is thus (*b*−*c*)^2^/4.

We now turn to the denominator of *z* ^2^. Suppose that in the *n* trios, there are exactly *n*_1_ where the parental mating type is *Aa*×*Aa*. Since the variance of *W* in any such trio is 1/2 and for all other mating types is 1/4, the variance of 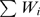 is *n*_1_/2+(*n*−*n*_1_)/4 = [2*n*_1_+(*n*−*n*_1_)]/4. This may be written, using the notation of the preceding paragraph, as (*b*+*c*)/4, because *b*+*c* is the total number of transmissions from heterozygous parents. It follows from the above that *z* ^2^ = (*b*−*c*)^2^/(*b*+*c*), and this is the standard qualitative TDT statistic of Spielman et al. [1].

Following a similar line of reasoning, if the variance of 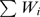 is estimated from the data, (as is done in the qualitative TDT procedure of Martin et al. [8], where such estimation is needed for non-trio data) the test of the hypothesis *α* = 0 in the regression (Equation 4) can be shown to be identical to the Martin et al. procedure [8].

#### Testing procedures best suited to various forms of data

The above considerations lead to a discussion of the data being analyzed. The original qualitative TDT of Spielman et al. [1] uses data only from children affected by some disease. Spielman et al. [1] also discuss an alternative to the qualitative TDT procedure when segregation distortion at the marker locus is suspected. In this alternative procedure, the proportion of transmissions of the *A* allele from heterozygous parents is compared not with 1/2, as in the original “standard” TDT, but with the corresponding proportion in nonaffected individuals. The analogues of “affected and not affected” in the quantitative TDT context might be “extreme and nonextreme phenotype values.” If the *y_i_* values in a quantitative TDT procedure are derived from a random sample, and if the null hypothesis is not true, one might expect extreme and nonextreme *y* values to tend to correspond to different *w* values. This might lead to a significant dependence of *w* on *y* (or equivalently of *y* on *w*). Thus for a random sample, a “slope” test such as those carried out by all quantitative TDT procedures described above appears to be appropriate. These procedures are analogous to the alternative qualitative TDT testing procedure of Spielman et al. [1].

Thus if the data analyzed concern either only extremely low or extremely high *y_i_* values (but not both), which might be thought of as corresponding to “affected” children, it might be more appropriate to carry out the qualitative TDT test that uses only data from such children. As shown above, this is identical to an “intercept” regression test. One may, if desired, carry out both this procedure and a (slope) quantitative TDT test, since in a regression procedure, the test of the slope and the test of the intercept in a regression line are independent. However, extreme phenotypes might well not have a normal distribution, so that those regression-based procedures that use *F* tests might be unreliable. The Rabinowitz and the Monks-Kaplan procedures are not subject to this problem. The information provided jointly by the qualitative TDT and the Rabinowitz or the Monks-Kaplan tests would show whether there is significant absolute linkage disequilibrium and also a significant change in linkage disequilibrium as the phenotypic value varies.

#### Population stratification in regression-based tests

The aim of the original qualitative TDT was to overcome potential problems arising from population stratification, and this was done by using the transmission values *w_i_*. By design, the Rabinowitz and Monks-Kaplan procedures also overcome population stratification problems, using the same approach. The situation is not, however, so straightforward for the regression models.

The simplest of the regression models considered above, namely Ab-Wthn, is a regression of *Y* on *w*. Once parental mating type information has been factored out of the regression, Ab-Orth, Al-Lin, and Al-Quad also have this property. The same is true of our suggested modification of the Ab-Dom procedure. It is thus tempting to argue that these regression procedures are immune to problems caused by population stratification, using the claim that it is sufficient to overcome stratification problems by using only *w* in the testing procedure. This conclusion is, however, not necessarily correct.

We illustrate this by considering an extreme case where the parental mating type *Aa*×*aa* occurs only in one stratum in the population, parental mating type *Aa*×*Aa* occurs only in another stratum, and parental mating type *Aa*×*AA* occurs only in a third stratum in the population. Suppose also that for reasons not connected with the marker locus, the null hypothesis mean phenotypes in the three strata are *μ*_1_ in the first stratum, *μ*_2_ in the second and *μ*_3_ in the third. Then of the models considered above, the Al-Lin model most closely reflects this situation. Suppose finally that for reasons having nothing to do with the marker locus, the three means—*μ*_1_, *μ*_2_,and *μ*_3_—are not all equal.

Suppose that despite this, the investigator uses the Ab-Wthn test, which in effect assumes equality of *μ*_1_, *μ*_2_, and *μ*_3_. The regression sum of squares (used in the numerator of the *F* ratio for this test) is
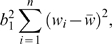
and the residual sum of squares (used in the numerator of the *F* ratio) is
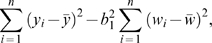
where *b*_1_ is the standard regression estimate of *β*_1_. If *μ*_1_ = *μ*_2_ = *μ*_3_, then under the null hypothesis, these sums of squares have expected values *σ* ^2^ and (*n*−2)*σ* ^2^ respectively, the corresponding mean squares both have expected values *σ* ^2^, and (assuming a normal distribution for the phenotype) the *F* statistic has the *F* distribution. If however *μ*_1_, *μ*_2_, and *μ*_3_ are not all equal, the null hypothesis mean values of these two mean squares are
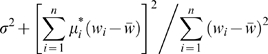
and
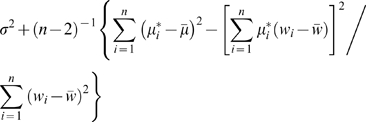
respectively. In these expressions all sums are taken over the *n* trios, 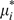 is the value of *μ* (either *μ*_1_, *μ*_2_, or *μ*_3_) appropriate for trio *i*, and 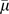 is the average of the 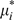 values. The term in square brackets in the first expression is clearly non-negative, and the term in square brackets in the second expression can be shown, via the Cauchy-Schwartz inequality, also to be non-negative. Thus the *F* statistic does not now have the *F* distribution when the null hypothesis (*β*_1_ = 0) is true, so that the type I error of the Ab-Wthn procedure will now not be at the assumed value. Simulations also show that it generally exceeds the assumed value. In this sense, and in this example, the Ab-Wthn procedure is not immune to population stratification. A similar observation was also reported by Yu et al. [9]. This is of course an extreme example, which might seldom arise in practice. It nevertheless shows that caution is needed in assuming that a test based on the *w_i_* values only is automatically immune to stratification problems for quantitative traits. It also indicates that the practitioner should make an assessment of which regression model most closely reflects the situation from which the data were obtained and use the testing procedure for that model.

#### Power considerations

Given the various quantitative TDT procedures, it is important to address the power comparisons between them. First it has to be noted that the power comparison between any two tests is only meaningful if both are “anchored” so as to have the same type I error. In the situation described in the previous paragraph, a power comparison between the Ab-Wthn and the Al-Lin test is not meaningful. (The Al-Lin test is valid in this situation, and has the assumed type I error.)

Suppose on the other hand that there is no population stratification associated with parental mating type and the Ab-Wthn test can be taken as appropriate. Then both the Ab-Wthn and Al-Lin procedures are valid tests, and the *F* ratios in the two tests both have the *F* distribution under the null hypothesis. The Al-Lin test will lose a small amount of power because of an unnecessary decrease in the residual number of degrees of freedom. It follows from all the above that no uniform statement about power can be made, and that the investigator has to use his/her judgment about the most appropriate test to use.

## Conclusions

These notes indicate that there are several matters that the investigator should keep in mind in his/her data analysis. First, as noted above, all the procedures described here test for changes in the phenotype value *Y* as a function of *W* (or equivalently changes in *W* as a function of *Y*). This implies that these procedures are best suited either to a random sample of data or to data only comprising both low and high values of the phenotype under discussion. If the data relate only to “extremely low” or to “extremely high” values of *Y*, the qualitative TDT procedure is perhaps more appropriate. Second, if the investigator suspects population stratification associated with mating types, careful consideration should be given to the test that is to be used. Third, the regression-based methods are more susceptible to departure from the normality assumption, but the Rabinowitz and Monks-Kaplan procedures are not. We suggest that users be cautious when interpreting results from different tests, especially when the distribution of the trait is non-normal.

In this brief review there are many topics that we have not covered. On the practical side, we have purposely not tried to recommend particular tests for specific kinds of data. This was not our goal, and in any case, would require considering a very large number of possible situations. Similarly, we have not discussed approaches to handle missing genotypes, although there are standard ways to do this [10]–[12]. On the theoretical side, we have not discussed the statistical theory behind the procedures described above. “Optimal” procedures often use score statistics, but the choice of the appropriate statistic relies on a choice of model that is felt best to describe the data. Next, we have considered only informative mating types, whereas some procedures use data from uninformative mating types, which may cause inflation of type I error rate if the phenotype distributions are different for different mating types. These and other theoretical questions will be taken up elsewhere.

## References

[1] Spielman RS, McGinnis RE, Ewens WJ (1993). Transmission test for linkage disequilibrium: the insulin gene region and insulin-dependent diabetes mellitus (IDDM).. Am J Hum Genet.

[2] Rabinowitz D (1997). A transmission disequilibrium test for quantitative trait loci.. Hum Hered.

[3] Monks SA, Kaplan NL (2000). Removing the sampling restrictions from family-based tests of association for a quantitative-trait locus.. Am J Hum Genet.

[4] Abecasis GR, Cardon LR, Cookson WO (2000). A general test of association for quantitative traits in nuclear families.. Am J Hum Genet.

[5] Allison DB (1997). Transmission-disequilibrium tests for quantitative traits.. Am J Hum Genet.

[6] Lange C, DeMeo DL, Laird NM (2002). Power and design considerations for a general class of family-based association tests: quantitative traits.. Am J Hum Genet.

[7] Laird NM, Horvath S, Xu X (2000). Implementing a unified approach to family based tests of association.. Genet Epidemiol.

[8] Martin ER, Kaplan NL, Weir BS (1997). Tests for linkage and association in nuclear families.. Am J Hum Genet.

[9] Yu J, Pressoir G, Briggs WH, Vroh Bi I, Yamasaki M (2006). A unified mixed-model method for association mapping that accounts for multiple levels of relatedness.. Nat Genet.

[10] Burdick JT, Chen WM, Abecasis GR, Cheung VG (2006). In silico method for inferring genotypes in pedigrees.. Nat Genet.

[11] Marchini J, Howie B, Myers S, McVean G, Donnelly P (2007). A new multipoint method for genome-wide association studies by imputation of genotypes.. Nat Genet.

[12] Li Y, Ding J, Abecasis GR (2007). Markov model for rapid haplotyping and genotype imputation in genome wide studies.. Am J Hum Genet.

